# Atorvastatin enhances radiosensitivity in hypoxia-induced prostate cancer cells related with HIF-1α inhibition

**DOI:** 10.1042/BSR20170340

**Published:** 2017-08-21

**Authors:** Bin Chen, Minguang Zhang, Dongwei Xing, Yu Feng

**Affiliations:** Radiology Department, Shanghai Municipal Hospital of Traditional Chinese Medicine, Shanghai University of Traditional Chinese Medicine, Shanghai 200071, China

**Keywords:** Atorvastatin, HIF-1α, prostate cancer, radiosensitivity

## Abstract

Hypoxia could enhance radioresistance in prostate cancer cells through up-regulating HIF-1α, which could be inhibited by statins in several cancer cells. However, this effect of statins in prostate cancer remains unclear. In the present study, we aim to investigate the effect of atorvastatin on HIF-1α expression and radiosensitivity in prostate cancer cells. The hypoxia-induced human prostate cancer PC3 cells were generated by incubating with 5% O_2_ for 24 h. The cell viability and apoptosis were respectively analyzed by cell counting kit-8 (CCK-8) assay and flow cytometry. The HIF-1α protein expression was assessed by Western blotting. HIF-1α expression in PC3 cells was significantly increased after incubating with 5% O_2_ for 24 h. The viability of hypoxia-induced PC3 cells was inhibited by a higher dose of irradiation than control cells. The viability of hypoxia-induced PC3 cells were inhibited by astorvastatin with a higher concentration than control cells. Astorvastatin reduced the HIF-1α protein expression in hypoxia-induced PC3 cells, and induced apoptosis of both control and hypoxia-induced cells with and without irradiation. Atorvastatin could enhance radiosensitivity in hypoxia-induced prostate cancer cells, which may be related with inhibition of HIF-1α protein.

## Introduction

Prostate cancer is the sixth leading cause of cancer deaths in males in the world [1]. Radiotherapy has been widely used as a curative treatment for prostate cancer [2]. In recent decades, radiotherapy in prostate cancer has undergone significant advances in technology and clinical practice, which contributes to a better cancer control outcome and less treatment morbidity than before [3,4]. However, prostate cancer radioresistance remains in a number of patients, leading to cancer relapse [5]. Radioresistance of prostate cancer may be mainly due to hypoxia in the center of tumors, which could induce a G_2_/M cell cycle arrest, inhibit apoptosis, and reduce the number of senescent cells [6]. Thus, hypoxia-induced prostate cancer would be a key point in overcoming the radioresistance in prostate cancer.

Statins are a class of lipid-lowing medication and have been recently found to be able to reduce the incidence and death due to cancers, including prostate cancer [7–9]. Amongst statins, atorvastatin has been one of the most commonly used ones in clinical practice [10,11]. It could promote radiosensitivity and apoptosis in PC3 prostate cancer cells [12]. However, the precise mechanism is unclear. A previous study found that hypoxia following irradiation could enhance radioresistance of prostate cancer cells through up-regulating HIF-1α [13]. Interestingly, HIF-1α could be inhibited by statins in a dose-dependent manner *in vitro* [6]. Thus, we suppose that atorvastatin may inhibit prostate cancer cells and enhance radiosensitivity of prostate cancers cells through inhibiting HIF-1α. In the present study, we will investigate this hypothesis with human prostate cancer PC3 cells.

## Methods

### Cell culture

Human prostate cancer PC3 cells were purchased from the Cell Centre, Institute of Biochemistry and Cell Biology, SIBS, CAS (Shanghai, China). All cells were maintained in RPMI-1640 medium (PAA Laboratories) supplemented with 10% FBS in a humidified incubator at 37°C with 5% CO_2_ and normoxic conditions.

### Western blotting

The protein expression of HIF-1α in PC3 cells was assessed by Western blotting. Cells from 80% confluent cultures were washed with PBS and resuspended in lysis buffer for 20 min at 4°C, pelleted at 10000 rpm for 10 min. Protein (50 μg) from each electrophoresis was added into 5× SDS buffer and was boiled to denaturation. Stacking gel (80 V) and 120 V of separating gel were used to separate proteins, which then were transferred on to PVDF membranes with 300 mA for 1 h. Low-fat milk (5%) was used to block the membranes for 12 h at 4°C, and then the membranes were probed for 8 h at 4°C with primary rabbit anti-human antibody against HIF-1α (ab82832, Abcam, U.K.) and goat anti-rabbit horseradish peroxidase–conjugated secondary antibodies (ab205718, Abcam, U.K.) for 2 h at room temperature. Peroxidase labeling was revealed by ECL Western blotting detection system (Thermo, U.S.A.).

### Cell counting kit-8 assay

The viability of PC3 cells was detected by cell counting kit-8 (CCK-8) assay. PC3 cells were seeded in 96-well plates for 12 h and then received irradiation or atorvastatin administration. On one hand, PC3 cells were cultured for 24 h after receiving different total dose of irradiation at the rate of 1.88G y/min, and then 10 µl CCK-8 solutions (Beyotime) were added into each well and incubated at 37°C for 2 h. On the other hand, after the cells were cultured with various concentrations of atorvastatin for 24 h at 37°C with 5% CO_2_, CCK-8 assay was also used. The absorbance was measured by a Microplate Reader (BioTek) at a wavelength of 450 nm.

### Flow cytometry

The apoptosis of PC3 cells was detected by flow cytometry. PC3 cells were washed with PBS and centrifuged (1000 rpm), and then were resuspended using Annexin-binding buffer. One hundred microliters of each sample were seeded in 96-well plates (2 × 10^5^–1 × 10^6^ cell/ml), and were incubated with 2.5 μl Annexin V-Alexa Fluor (FA101-02, TransGen Biotech, Beijing, China) at 37°C for 20–30 min. One microliter of propidium iodide (100 μg/ml) and 400 μl of Annexin-binding buffer was added into each well. And then those cells were analyzed on a flow cytometer (BD Biosciences, San Jose, CA, U.S.A.) with a 488-nm laser. The emissions were captured at 530 and 575 nm, respectively.

### Statistical analysis

Student’s *t* test was used to assess the difference between the two groups. Results were shown as mean ± S.D. *P*<0.05 was considered to be statistically significant. Statistical analyses were performed using IBM SPSS ver. 21.0 software (IBM Co., Armonk, NY, U.S.A.).

## Results

### Hypoxia induces protein expression of HIF-1α in PC3 cells

**Figure 1:**
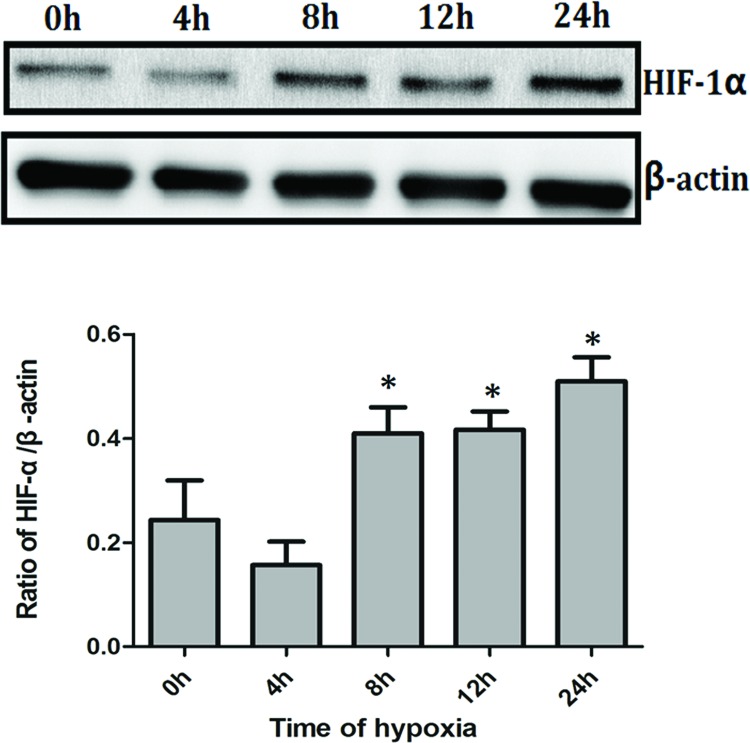
Hypoxia induces protein expression of HIF-1α in PC3 cells PC3 cells were incubated with 5% O_2_ for 4, 8, 12, and 24 h, respectively. HIF-1α protein expression in PC3 cells was increased after incubating for 8 h and was highest after 24 h. *P<0.05 when compared with 0 h.

PC3 cells were incubated with 5% O_2_ for 4, 8, 12, and 24 h, respectively. The proteins expression of HIF-1α in PC3 cells were then assessed by Western blotting. HIF-1α expression in PC3 cells was increased after incubating for 8 h, and enhanced in a time-dependent manner of hypoxia. HIF-1α expression in PC3 cells was highest after incubating for 24 h (Figure 1).

### Radiosensitivity of hypoxia-induced PC3 cells is decreased

**Figure 2:**
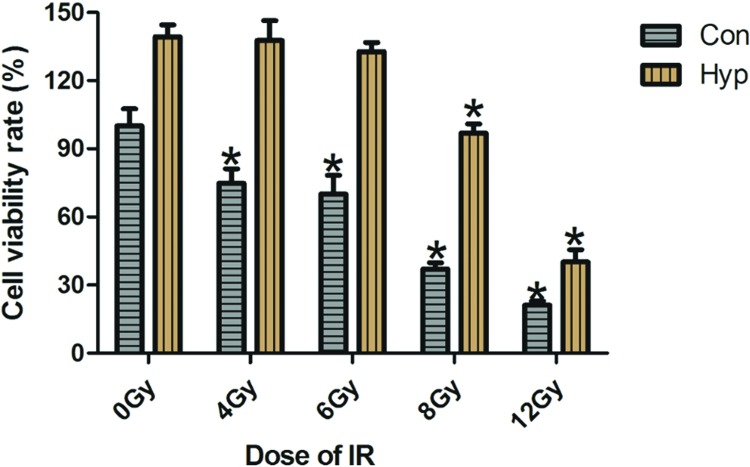
Radiosensitivity in hypoxia-induced PC3 cells is decreased. The hypoxia-induced PC3 cells were obtained by incubating with 5% O_2_ for 24 h, and then received irradiation with γ rays in doses of 4, 6, 8, and 12 Gy, respectively. Twenty-four hours after irradiation, the viability of these cells was detected by CCK-8. Control PC3 cells could be inhibited by irradiation with 4 Gy, whereas hypoxia-induced PC3 cells were inhibited by 8 Gy. Abbreviations: Con, control PC3 cell; Hyp, hypoxia-induced PC3 cell; IR, irradation.

The hypoxia-induced PC3 cells were obtained by incubating with 5% O_2_ for 24 h, since HIF-1α expression in PC3 cells was highest after incubating for 24 h. These hypoxia cells respectively received irradiation with γ rays at doses of 4, 6, 8, and 12 Gy at the rate of 1.88 Gy/min. After irradiation, the viability of these cells was detected by CCK-8 assay. Control PC3 cells could be inhibited by irradiation with 4 Gy, whereas hypoxia-induced PC3 cells were inhibited by 8 Gy (Figure 2). Thus, hypoxia-induced PC3 cells were less radiosensitive than the control ones.

### Atorvastatin toxicity test in control and hypoxia-induced PC3 cells

**Figure 3:**
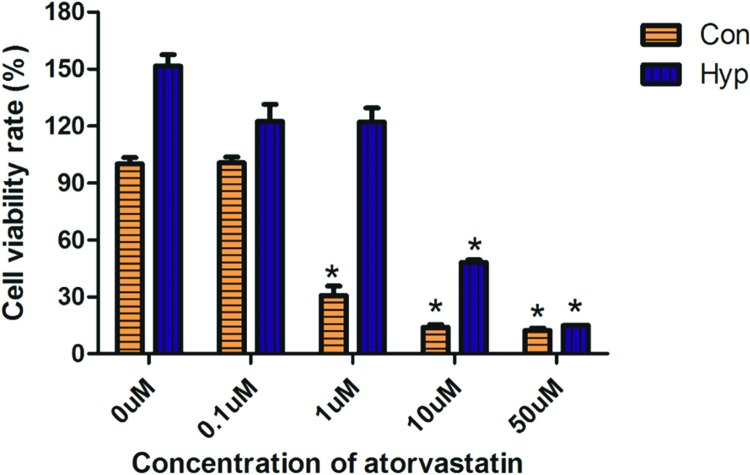
Atorvastatin inhibits hypoxia-induced PC3 cells Atorvastatin at concentrations of 0.1, 1, 5, 10, or 50 μM was respectively cultured with control and hypoxia-induced PC3 cells. After 24 h, the viability of these cells was detected by CCK-8 assay. The viability of control PC3 cells became reduced by atorvastatin at a concentration of 1 μM, whereas hypoxia-induced PC3 cells were inhibited with a concentration of 10 μM. *P<0.05 when compared with 0 μM. Abbreviations: Con, control PC3 cell; Hyp, hypoxia-induced PC3 cell.

A range from 0.1 to 50 μM of atorvastatin was used. Atorvastatin at concentrations of 0.1, 1, 5, 10, or 50 μM were respectively cultured with control and hypoxia-induced PC3 cells. After 24 h, the viability of these cells was detected by CCK-8 assay. The viability of control PC3 cells could be reduced by atorvastatin at a concentration of 1 μM, whereas hypoxia-induced PC3 cells were inhibited at a concentration of 10 μM (Figure 3). Thus, a concentration of 1 μM may be appropriate in inhibiting PC3 cells. This result suggests that atorvastatin could inhibit PC3 cells in a dose-dependent manner. But hypoxia-induced PC3 cells were less sensitive for atorvastatin than the control ones.

### Atorvastatin inhibits protein expression of HIF-1α in hypoxia-induced PC3 cells

**Figure 4:**
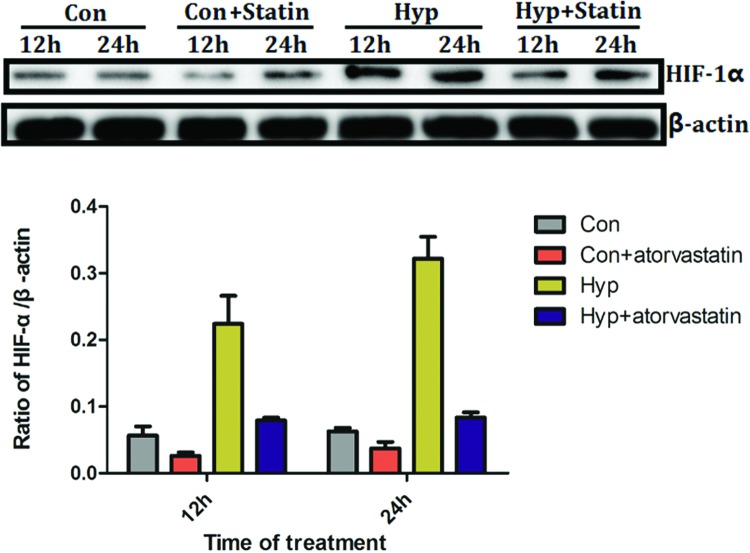
Atorvastatin inhibits protein expression of HIF-1α in hypoxia-induced PC3 cells Atorvastatin at a concentration of 1 μM was cultured with control and hypoxia-induced PC3 cells for 12 and 24 h, respectively. In hypoxia-induced PC3 cells, the protein expression of HIF-1α was increased, which could be inhibited by atorvastatin for 12 and 24 h. Abbreviations: Con, control PC3 cell; Hyp, hypoxia-induced PC3 cell.

Atorvastatin at a concentration of 1 μM was cultured with control and hypoxia-induced PC3 cells for 12 and 24 h, respectively. In hypoxia-induced PC3 cells, the protein expression of HIF-1α was increased, which could be inhibited by atorvastatin for 12 h (Figure 4). Thus, atorvastatin could inhibit the HIF-1α expression in hypoxia-induced PC3 cells.

### Atorvastatin enhances radiosensitivity in PC3 cells

**Figure 5:**
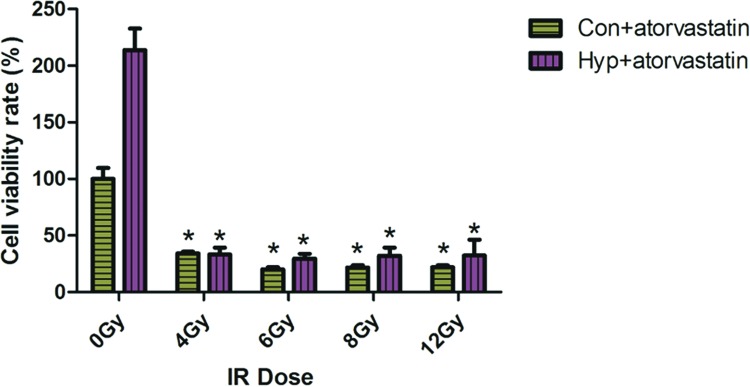
Atorvastatin inhibits the viability of hypoxia-induced PC3 cells after irradiation Control and hypoxia-induced PC3 cells received irradiation with 6 Gy, atorvastatin, or irradiation (6 Gy) + atorvastatin for 24 h. After culturing with atorvastatin, the viability of both control and hypoxia-induced PC3 cells was decreased after being radiated with 4 Gy. *P<0.05 when compared with 0 Gy. Abbreviations: Con, control PC3 cell; Hyp, hypoxia-induced PC3 cell.

**Figure 6:**
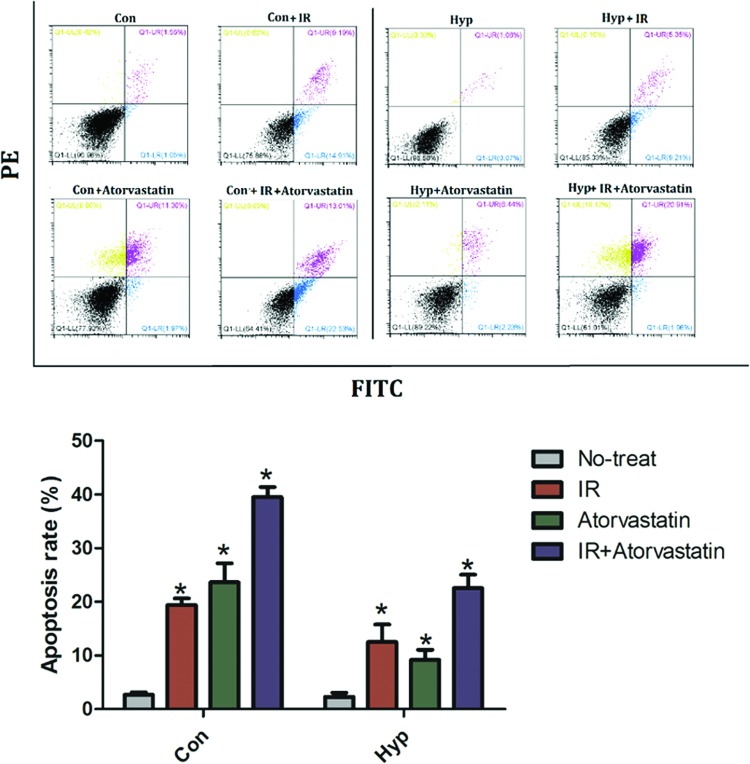
Atorvastatin enhances the apoptosis of hypoxia-induced PC3 cells after irradiation Control and hypoxia-induced PC3 cells received irradiation with 6 Gy, atorvastatin, or irradiation (6 Gy) + atorvastatin for 24 h. Atorvastatin increased the apoptosis in both control and hypoxia-induced PC3 cells, compared with controls. The irradiation-induced apoptosis in control or hypoxia-induced PC3 cells was also increased by atorvastatin, when compared with those that received irradiation alone. *P<0.05 when compared with control. Abbreviations: Con, control PC3 cell; Hyp, hypoxia-induced PC3 cell; IR, irradiation.

Control and hypoxia-induced PC3 cells received irradiation with 6 Gy, atorvastatin (1 μM), or irradiation (6 Gy) + atorvastatin (1 μM) for 24 h. After culturing with atorvastatin, the viability of both control and hypoxia-induced PC3 cells was decreased after radiating with 4 Gy (Figure 5). Moreover, atorvastatin increased the apoptosis in both control and hypoxia-induced PC3 cells, compared with controls. The irradiation-induced apoptosis in control or hypoxia-induced PC3 cells was also increased by atorvastatin, when compared with those that received irradiation alone (Figure 6). These suggested that atorvastatin could not only induce apoptosis in control and hypoxia-induced PC3 cells, but also could enhance radiosensitivity in these cells.

## Discussion

In our study, we found that hypoxia-induced prostate cancer PC3 cells were less radiosensitive than control cancer cells. Hypoxia could induce the expression of HIF-1α in PC3 cells. Atorvastatin could inhibit HIF-1α protein level in hypoxia-induced PC3 cells, induce apoptosis in both control and hypoxia-induced cells with and without irradiation. Our results suggest that atorvastatin could enhance radiosensitivity of prostate cancer cells through inhibiting HIF-1α protein level.

Our study found that hypoxia could induce prostate cancer cells to resist irradiation. Hypoxia is a characteristic of prostate cancer microenvironment due to a quick outstrip blood supply of tumor [14]. And chronic hypoxia subregions are the key point of radioresistence [15]. Our results were consistent with the previous findings that hypoxia induces radioresistence to prostate cancer, and suggested that our model of hypoxia-induced prostate cancer cells was successfully constructed. Since hypoxia-induced prostate cancer cells were significantly inhibited from the dose of 6 Gy irradiation, 6 Gy was used in testing the effect of atorvastatin on the radiosensitivity of prostate cancer.

Hypoxia-induced signaling, particularly HIF-1α, could promote the progression of prostate cancer through regulating various genes’ expressions, which are associated with angiogenesis, epithelial-to-mesenchymal transition, metastasis, survival, proliferation, metabolism, sternness, hormone-refractory progression, and therapeutic resistance [16]. Thus, HIF-1α regulates proliferation, apoptosis, and migration of PC3 cells [17]. In our study, HIF-1α expression was induced by hypoxia in human prostate cancer, confirming findings of previous studies [13] and suggesting a key role of HIF-1α in prostate cancer within a hypoxia microenvironment.

Moreover, our study found that atorvastatin could inhibit protein expression of HIF-1α in human prostate cancer in a dose-dependent manner, suggesting a potentially antitumor effect of statins in prostate cancer. Since HIF-1α may promote radioresistance of prostate cancer [18], we further investigate the effect of atorvastatin on radiosensitivity of prostate cancer cells. In our study, after culturing with atorvastatin, the viability was decreased, whereas the apoptosis was increased in both control and hypoxia-induced prostate cancer cells. Thus, atorvastatin could increase the radiosensitivity of not only control prostate cancer cells but also hypoxia-induced cells. Therefore, our study suggested that atovastatin could increase radiosensitivity in prostate cancer cells, especially hypoxia-induced prostate cancer cells, through inhibition of HIF-1α.

In conclusion, atorvastatin could enhance radiosensitivity of hypoxia-induced prostate cancer cells, which may be related with inhibition of HIF-1α protein. Our findings may provide a novel way to overcome the obstacles of cancer radioresistance, and contribute to further study on the treatment of radioresistent prostate cancer.

## Abbreviations

CCK-8: cell counting kit-8
HIF-1a: hypoxia inducible factor
SIBS: Shanghai Institutes for Biological Sciences
CAS: Chinese Academy of Sciences
RPMI-1640: Roswell Park Memorial Institute-1640
SPSS: Statistical Product and Service Solutions
